# Supplementary material for: Influence of Citrus Scion/Rootstock Genotypes on Arbuscular Mycorrhizal Community Composition under Controlled Environment Condition
Source: Plants (Basel). 2020 Jul 16;9(7):901. doi: 10.3390/plants9070901 (PMC7412222; doi:10.3390/plants9070901)

Fig. S1 Configurations of six citrus scion/rootstock genotype combinations from the same field in a greenhouse. *Poncirus*/*Poncirus* (*Poncirus* (*Poncirus trifoliate*) grafted onto *Poncirus*), *Citrange*/*Citrange* (*Citrange* (*Citrus sinensis*×*Poncirus trifoliate*) grafted onto *Citrange*), *Mandarin*/*Poncirus* (*Mandarin* (*Citrus reticulate*) grafted onto *Poncirus*), *Pummelo*/*Poncirus* (*HB pummelo* (*Citrus grandis*) grafted onto *Poncirus*), *Orange/Poncirus* (*Newhall sweet orange* (*Citrus sinensis*) grafted onto *Poncirus*), *Orange/Citrange* (*Newhall sweet orange* grafted onto *Citrange*).

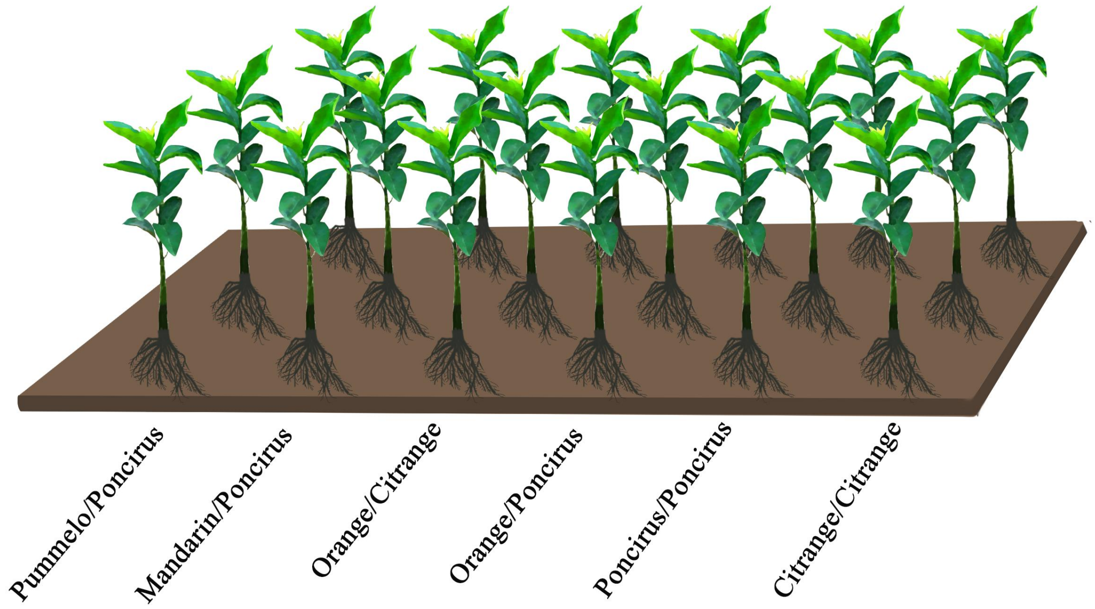

Fig. S2 Rarefaction curve of Sobs Index of 18 root samples from 6 citrus scion/rootstock genotype combinations.

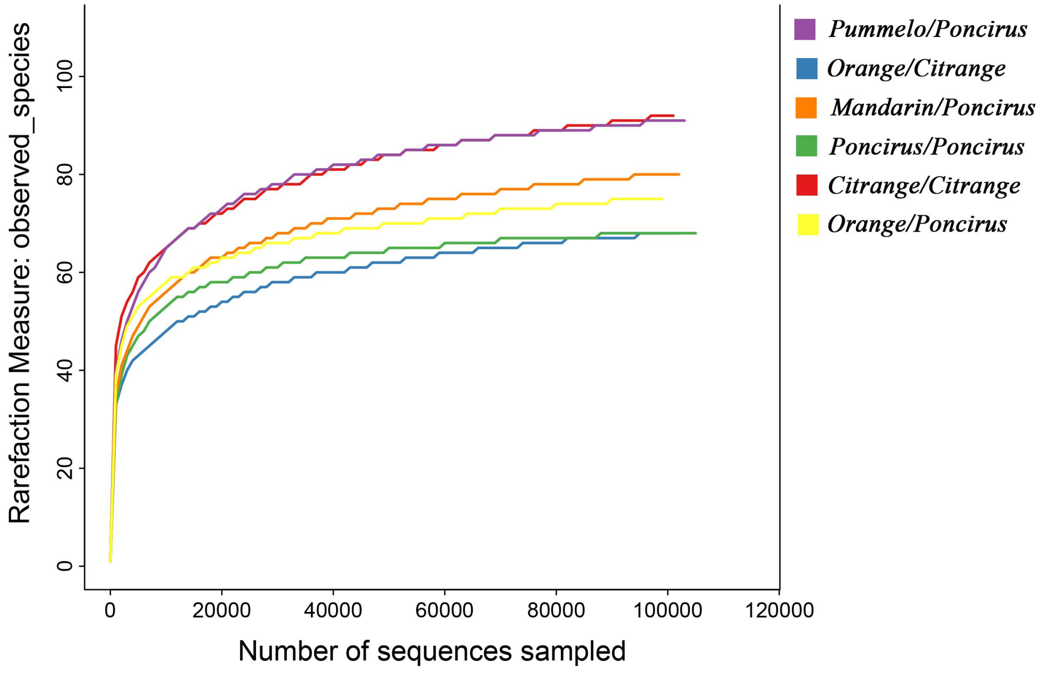

Supplement: Supplementary file 1 [file plants-09-00901-s001.zip › Supplymetry figures.pdf]
